# Perfect Controlled Multi-Output Teleportation of Single-Qubit States via a High-Dimensional Partially Entangled Channel

**DOI:** 10.3390/e28060648

**Published:** 2026-06-08

**Authors:** Nueraminaimu Maihemuti, Yimamujiang Aisan, Jiayin Peng, Jiangang Tang

**Affiliations:** School of Mathematics and Statistics, Kashi University, Kashi 844000, Chinagreatxyzme@163.com (Y.A.); jg-tang@163.com (J.T.)

**Keywords:** perfect quantum teleportation, controlled multi-output teleportation, high-dimensional partially entangled channel, asymmetric joint measurement, single-qubit state

## Abstract

In practical quantum communication, quantum channels are inevitably affected by noise and decoherence, leading to their degradation into non-maximally entangled or even mixed states. As a result, conventional quantum teleportation schemes based on non-maximally entangled channels are inherently probabilistic and cannot simultaneously achieve unit fidelity and unit success probability. To address this issue, we exploit the structural degrees of freedom of high-dimensional partially entangled channels and construct an asymmetric joint measurement basis matched to the Schmidt-coefficient distribution of the channel, thereby proposing a controlled multi-output perfect quantum teleportation scheme. First, based on a three-dimensional partially entangled five-qutrit channel, a controlled two-output teleportation model for unknown single-qubit states is established. Perfect transmission with both unit fidelity and unit success probability is achieved through the controller’s projective measurement, the sender’s asymmetric joint measurement, and the receivers’ corresponding local recovery operations. On this basis, the scheme is generalized to arbitrary *d*-dimensional partially entangled channels and further extended from the two-output configuration to the multi-output scenario. Our analysis shows that, when the two largest Schmidt coefficients of the channel are equal, deterministic perfect teleportation with both unit fidelity and unit success probability can still be achieved using non-maximally entangled resources. The proposed scheme is more consistent with realistic quantum communication environments and provides a theoretical foundation for efficient and controllable quantum information distribution in complex quantum networks.

## 1. Introduction

Quantum teleportation is one of the most fundamental and important protocols in quantum information science. Since Bennett et al. first proposed the transmission of an unknown quantum state using pre-shared entanglement and classical communication [1], quantum teleportation has gradually developed into a core technique for quantum repeaters, quantum networks, and distributed quantum computation [2,3,4]. In the standard quantum teleportation protocol, two communicating parties usually pre-share a maximally entangled state as an ideal quantum channel. Through Bell-state joint measurements, classical communication, and the corresponding unitary recovery operations, perfect quantum teleportation with both fidelity and success probability equal to one can be achieved under ideal conditions [5,6,7]. However, in practical physical implementations, quantum systems inevitably interact with both internal and external environments. As a result, environmental noise, decoherence, channel loss, and device imperfections may degrade maximally entangled channels into partially entangled states or mixed states [8,9,10]. In this case, directly applying conventional quantum teleportation schemes usually makes it difficult to simultaneously maintain unit fidelity and unit success probability. The collapsed state obtained by the receiver often contains amplitude distortions associated with the nonuniform Schmidt coefficients of the channel. Therefore, the study of quantum teleportation based on non-maximally entangled or partially entangled channels has become an important issue in practical quantum communication.

To address this problem, various quantum teleportation schemes based on non-maximally entangled resources have been proposed [11,12,13]. Existing studies on noisy channels or partially entangled channels have shown that teleportation performance usually decreases due to channel degradation, insufficient entanglement, and decoherence effects. In noisy channels, these effects are mainly reflected in the reduction of teleportation fidelity [14,15]; whereas in conventional schemes based on partially entangled channels, the recovery of the target state with unit fidelity can usually be achieved only conditionally, and the total success probability remains less than one [16,17,18]. To realize the recovery of an unknown quantum state with unit fidelity, probabilistic quantum teleportation schemes were subsequently proposed. In such schemes, auxiliary particles, generalized measurements, or conditional operations are usually introduced. For example, the receiver may introduce an auxiliary particle initially prepared in the state |0〉, perform a specific transformation, and then measure the auxiliary particle. When the measurement result of the auxiliary particle is |0〉, the teleportation succeeds; when the result is |1〉, the teleportation fails. Therefore, conventional quantum teleportation schemes based on partially entangled channels generally have a success–failure branch structure: the teleported state can be recovered with fidelity one only when certain specific measurement outcomes are obtained, while the total success probability remains less than one [19,20,21,22]. This success probability is closely related to the channel coefficients and is usually limited by the smallest coefficient of the partially entangled channel. Therefore, unless the channel reduces to a maximally entangled state, the success probability generally cannot reach one. To improve the total success probability, recoverable or information-preserving quantum teleportation schemes were further developed [23,24,25,26]. The core idea of these schemes is that, even if a teleportation attempt fails, the information of the unknown quantum state is not completely destroyed; therefore, the sender and the receiver can repeat the teleportation process with the aid of additional quantum channels. As the number of repeated transmissions increases, the cumulative success probability can be gradually enhanced and, under ideal conditions, approach one. Nevertheless, such schemes still rely on repeated transmission processes and additional entangled resources. They can only improve the overall success probability asymptotically, but do not eliminate the intrinsic success–failure branch structure of probabilistic teleportation in a single transmission process. Therefore, they still cannot realize deterministic perfect quantum teleportation in a truly single-shot sense. To overcome this limitation, in addition to considering the entangled resource itself, it is necessary to further focus on the design of the measurement basis. In the teleportation process, the measurement operation plays a key role: different joint measurement bases lead to different forms of collapsed states and directly determine whether the receiver can achieve exact recovery through the corresponding unitary operations. In particular, when a partially entangled channel is used, a conventional measurement basis usually causes the collapsed state obtained by the receiver to contain amplitude distortions dependent on the channel coefficients, so that teleportation can succeed only probabilistically. Recent studies have shown that the realization of perfect quantum teleportation depends not only on the entangled resource itself, but also on whether the measurement basis can be matched with the Schmidt-coefficient structure of the partially entangled channel [27]. If an asymmetric joint measurement basis adapted to the structure of the partially entangled channel can be constructed, it may be possible to compensate for the amplitude distortions caused by the nonuniform channel coefficients, so that all possible measurement outcomes can be corrected by appropriate unitary recovery operations. In this way, deterministic perfect quantum teleportation can be realized without postselection, auxiliary-particle-assisted filtering, or repeated transmission. This indicates that non-maximally entangled resources do not necessarily lead to degraded teleportation performance, and that a proper measurement design may provide an important route to overcoming the limitations of conventional probabilistic quantum teleportation.

Inspired by this idea, a key question naturally arises: can this measurement-matched perfect teleportation strategy be further extended to more complex quantum communication networks? It is well known that, as quantum networks gradually evolve from traditional point-to-point structures to multi-user, multi-node, and distributed cooperative structures, quantum teleportation is required not only to realize the reliable transmission of unknown quantum states, but also to meet more complex communication requirements, such as security control, multi-user cooperation, and quantum information distribution. In this context, controlled quantum teleportation has gradually become an important research direction in quantum communication [28]. Karlsson and Bourennane first proposed the controlled quantum teleportation model [29], pointing out that the receiver can recover the target quantum state only when the controller participates and announces the measurement result. In this way, traditional bipartite quantum teleportation is extended to multipartite authorized quantum teleportation, thereby enhancing the security and controllability of quantum communication at the protocol level. Subsequently, extensive studies have been carried out on the theoretical models, implementation mechanisms, and resource optimization of controlled quantum teleportation [30,31,32]. Under nonideal entangled resources, Jiang et al. investigated bidirectional controlled quantum teleportation based on non-maximally entangled states and analyzed the influence of entanglement degradation on teleportation performance [33]. Singh et al. and Peng et al. further extended controlled teleportation to multi-directional transmission and cyclic communication structures [34,35,36]. However, most existing controlled teleportation schemes mainly focus on single-output transmission, while controlled multi-output quantum teleportation based on non-maximally entangled high-dimensional channels remains insufficiently studied.

On the other hand, with the continuous expansion of quantum networks, multi-output quantum teleportation has become increasingly important. Compared with traditional single-output teleportation, multi-output schemes can distribute quantum information to multiple receivers simultaneously, thereby improving resource utilization and quantum information distribution efficiency in quantum networks [37,38,39]. Ishizaka and Hiroshima proposed the theoretical framework of port-based quantum teleportation, which laid an important foundation for the study of multi-output quantum teleportation [40,41]. In 2017, Yu et al. realized a quantum broadcasting and multi-output teleportation scheme based on a four-qubit cluster state, enriching the physical implementation approaches of multi-output transmission [42]. In 2021, Yu et al. completed an experimental verification of multi-output teleportation of different quantum information on the IBM quantum platform, confirming the feasibility of such schemes on real quantum hardware [43]. In 2023, Kumar studied multi-output quantum teleportation using optimal quantum resources, providing a reference for reducing protocol resource costs [44]. Subsequently, Yan et al. reported a deterministic multi-user quantum teleportation network for continuous-variable polarization states, further highlighting the importance of scalable multi-user teleportation networks for quantum information distribution [45]. In recent years, Peng’s group and related studies have further combined the idea of multi-output transmission with controlled communication, proposing schemes such as bidirectional multi-output quantum communication, multilayer controlled multi-output quantum teleportation, and multi-output nondestructive controlled quantum teleportation based on partially entangled channels [46,47,48]. These works show that multi-output quantum communication will play an important role in future quantum networks. However, most existing multi-output schemes rely on maximally entangled states, or can only realize probabilistic transmission under non-maximally entangled conditions, making it difficult to achieve both unit fidelity and unit success probability simultaneously. Therefore, how to realize deterministic perfect controlled multi-output quantum teleportation under non-maximally entangled resources remains a challenging research problem.

The development of high-dimensional quantum systems provides new possibilities for solving the above problem. Compared with traditional two-dimensional quantum systems, high-dimensional quantum systems have richer state-space structures, larger Hilbert spaces, and more flexible measurement-design freedom [49,50]. These features enable high-dimensional quantum channels not only to carry richer quantum information, but also to provide greater freedom for constructing nonstandard joint measurement bases and realizing complex quantum-state transformations [51,52]. In particular, high-dimensional entangled channels can break through the constraints of traditional equal-dimensional Bell-type measurements, allowing researchers to construct more adaptive asymmetric joint measurement bases according to the Schmidt structure and entanglement distribution of the channel, thus making it possible to compensate for the amplitude distortion caused by partially entangled channels [53,54]. Existing studies have shown that high-dimensional entangled channels can be used to transmit low-dimensional unknown quantum states. For example, Zhan et al. proposed a scheme for teleporting an unknown single-qubit state via an arbitrary high-dimensional entangled state [55]; Song et al. further constructed a multi-output controlled teleportation model based on arbitrary high-dimensional entangled states [56]; Lei et al. studied several controlled bidirectional and multiparticle quantum teleportation schemes based on arbitrary high-dimensional entangled states [57,58,59], continuously expanding the application scenarios of high-dimensional quantum teleportation. However, most current high-dimensional transmission schemes still rely on ideal maximally entangled channels and therefore cannot fully adapt to realistic noise environments and channel degradation scenarios. Studies on non-maximally or partially entangled high-dimensional channels are mostly limited to traditional probabilistic transmission frameworks. Therefore, how to fully exploit the structural freedom of high-dimensional quantum channels, design asymmetric joint measurement strategies precisely matched to partially entangled channels, and realize deterministic perfect quantum teleportation with both unit fidelity and unit success probability under non-maximally entangled resources remains a key problem to be solved.

Based on the above research background, this paper combines the advantages of controlled quantum teleportation, multi-output quantum transmission, and high-dimensional quantum channels within a unified framework, and proposes a controlled multi-output perfect quantum teleportation scheme for unknown single-qubit states based on high-dimensional partially entangled channels. By constructing an asymmetric joint measurement basis matched with the Schmidt coefficients of the channel, the collapsed states at the receivers’ ends can preserve the linear structure of the unknown coefficients, so that all authorized receivers can perfectly recover the target quantum states with the participation of the controller. Compared with existing schemes, the proposed scheme is not only applicable to non-maximally entangled quantum channels and thus more consistent with practical physical conditions in quantum communication, but also supports controlled and multi-output quantum transmission. It can realize the synchronous recovery of quantum states by multiple receivers in a single transmission process, thereby effectively reducing the complexity and asynchrony caused by traditional multiple transmissions. In addition, by fully exploiting the advantages of high-dimensional quantum channels in state-space structure and measurement design, the proposed scheme realizes deterministic perfect quantum teleportation with both fidelity and success probability equal to 1. This unified framework, which integrates non-maximally entangled resources, high-dimensional quantum channels, controlled transmission, multi-output communication, and deterministic perfect teleportation, provides a feasible approach for efficient and controllable quantum information distribution in complex quantum networks.

The rest of this paper is organized as follows. Section 2 introduces the relevant preliminaries and constructs the required high-dimensional partially entangled quantum channels. Section 3 presents a controlled two-output single-qubit teleportation scheme based on a three-dimensional partially entangled state. Section 4 generalizes the scheme to the case of a general *d*-dimensional partially entangled channel. Section 5 further extends the scheme to controlled multi-output single-qubit teleportation under high-dimensional partially entangled channels. Finally, Section 6 summarizes the paper and discusses the results.

## 2. Preliminary and Construction of Quantum Channels

To describe our schemes more clearly, this section requires a review of some basic concepts, terminology, and the like. An arbitrary single-qubit state in 2-dimensional Hilbert space is a superposition of the ground state |0〉 and the polarized state |1〉, which can be written as

(1) $$\begin{array}{c} |\varphi \rangle =\alpha |0\rangle +\beta |1\rangle , \end{array}$$

where complex numbers α and β satisfy the normalization condition ${\left|\alpha \right|}^{2}+{\left|\beta \right|}^{2}=1$. Similarly, an arbitrary single-qudit state in *d*-dimensional Hilbert space can be represented as

(2) $$\begin{array}{c} |\psi \rangle =\beta_{0}|0\rangle +\beta_{1}|1\rangle +\cdots +\beta_{d-1}|d-1\rangle , \end{array}$$

where $\beta_{0},\beta_{1},\cdots ,\beta_{d-1}$ are complex numbers with $\sum_{j=0}^{d-1}{\left|\beta_{j}\right|}^{2}=1$. Specifically, when d=3, we obtain a single-qutrit state in three-dimensional Hilbert space, which is given as

(3) $$\begin{array}{c} |\psi^{\prime }\rangle =x|0\rangle +y|1\rangle +z|2\rangle \end{array}$$

with ${\left|x\right|}^{2}+{\left|y\right|}^{2}+{\left|z\right|}^{2}=1$. The generalized Bell states (GBS) of *d*-dimensional quantum systems are

(4) $$\begin{array}{c} |\Psi_{st}\rangle =\frac{1}{\sqrt{d}}\sum_{j=0}^{d-1}e^{2\pi ijs/d}|j\rangle |j⊕t\rangle , \end{array}$$

where the symbol ⊕ denotes addition modulo *d*, and s,t=0,1,⋯,d−1 are used to label the $d^{2}$ orthogonal GBS. |0〉, |1〉, ⋯, and |d−1〉 are the *d* eigenvectors of the measuring basis (MB) $Z_{d}$. The $d^{2}$ unitary transformations $U_{uv}$ (u,v=0,1,⋯,d−1) can transfer one of the generalized Bell states into each other:

(5) $$\begin{array}{c} U_{uv}=\sum_{j=0}^{d-1}e^{2\pi iju/d}|j⊕v\rangle \langle j|. \end{array}$$

Another unbiased basis $X_{d}$ which has *d* eigenvectors can be written as ${\{|0\rangle }_{x}{,|1\rangle }_{x},\cdots ,{|r\rangle }_{x},\cdots ,$${|d-1\rangle }_{x}\}$:

(6) $$\begin{array}{c} {|r\rangle }_{x}=\frac{1}{\sqrt{d}}\sum_{j=0}^{d-1}e^{2\pi ijr/d}|j\rangle , \end{array}$$

where r∈{0,1,⋯,d−1}. The two unbiased bases have the relation ${|\langle k|r\rangle }_{x}{|}^{2}=1/d$. Here |k〉 is an eigenvector of the MB $Z_{d}$ and ${|r\rangle }_{x}$ is an eigenvector of the MB $X_{d}$.

A generalization of the Hadamard gate to d-dimensional quantum systems (also known as the quantum Fourier transform) plays an important role in quantum information processing [4,60].

(7) $$\begin{array}{c} H:=\frac{1}{\sqrt{d}}\sum_{k,l=0}^{d-1}\exp\left\{\frac{2\pi i}{d}kl\right\}|k\rangle \langle l|. \end{array}$$

This operator reduces to the quantum Fourier transform when $d=2^{n}$, in which case it acts on *n* qubits. Here we treat it as a fundamental gate acting on a single qudit, analogous to the Hadamard gate for a single qubit.

In 2002, Karimipour and Bahraminasab [61] introduced the generalized controlled-NOT (GCNOT) gate in *d*-dimensional quantum systems, which is defined as:

(8) $$\begin{array}{c} \mathrm{GCNOT}(|k\rangle ,|l\rangle )=|k\rangle |k⊕l\rangle ,\;\;k,l\in \{0,1,\cdots ,d-1\}, \end{array}$$

where the first qudit and the second qudit are the control qudit and the target qudit respectively.

Generalized Hadamard gate and generalized controlled-NOT gate are commonly used fundamental quantum gates. As an example, we use these two types of gates to construct a quantum entangled state ${|\varphi \rangle }_{12345}$. The specific structure is as follows: The input state ${|\varphi \rangle }_{12345}$ is composed of the product state of the ground states of five qudits, i.e., $|\varphi_{0}\rangle_{12345}={|00000\rangle }_{12345}$, where the subscript numbers represent different qudits. Firstly, qudit 1 is fed into a generalized Hadamard gate, and then generalized controlled-NOT gates are applied to qudit pairs (1,2) and (1,4) respectively, where qudit 1 serves as the control qudit and qudits 2 and 4 serve as the target qudits. These two quantum gates convert the input state $|\varphi_{0}\rangle_{12345}$ into

(9) $$\begin{array}{c} |\varphi_{1}\rangle_{12345}=\frac{1}{\sqrt{d}}\sum_{k=0}^{d-1}{|k,k,0,k,0\rangle }_{12345}. \end{array}$$

Secondly, by feeding qudits 1 and 4 into generalized Hadamard gates respectively, the state $|\varphi_{1}\rangle_{12345}$ becomes

(10) $$\begin{array}{c} |\varphi_{2}\rangle_{12345}=\frac{1}{\sqrt{d^{3}}}\sum_{k=0}^{d-1}{|k\rangle }_{1}\left[\sum_{j=0}^{d-1}\exp\left\{\frac{2\pi i}{d}kj\right\}{|j,0\rangle }_{23}\right]\left[\sum_{l=0}^{d-1}\exp\left\{\frac{2\pi i}{d}kl\right\}{|l,0\rangle }_{45}\right]. \end{array}$$

Finally, by feeding qudit pairs (2,3) and (4,5) into generalized controlled-NOT gates respectively, where qudits 2 and 4 serve as control qudits and qudits 3 and 5 serve as target qudits, the quantum state $|\varphi_{2}\rangle_{12345}$ becomes

(11) $$\begin{array}{c} {|\varphi \rangle }_{12345}=\frac{1}{\sqrt{d^{3}}}\sum_{k=0}^{d-1}{|k\rangle }_{1}\left[\sum_{j=0}^{d-1}\exp\left\{\frac{2\pi i}{d}kj\right\}{|jj\rangle }_{23}\right]\left[\sum_{l=0}^{d-1}\exp\left\{\frac{2\pi i}{d}kl\right\}{|ll\rangle }_{45}\right]. \end{array}$$

A more general form of the entangled state ${|\varphi \rangle }_{12345}$ is the following partially entangled five-qudit state $|\varphi^{\prime }\rangle_{12345}$:

(12) $$\begin{array}{c} |\varphi^{\prime }\rangle_{12345}=\frac{1}{\sqrt{d}}\sum_{k=0}^{d-1}{|k\rangle }_{1}\left[\sum_{j=0}^{d-1}a_{kj}\exp\left\{\frac{2\pi i}{d}kj\right\}{|jj\rangle }_{23}\right]\left[\sum_{l=0}^{d-1}b_{kl}\exp\left\{\frac{2\pi i}{d}kl\right\}{|ll\rangle }_{45}\right], \end{array}$$

where real numbers $a_{kj},b_{kl}$ (k,j,l∈{0,1,⋯,d−1}) satisfy the normalization conditions $\sum_{j=0}^{d-1}a_{kj}^{2}=1$ and $\sum_{l=0}^{d-1}b_{kl}^{2}=1$ for any k∈{0,1,⋯,d−1}.

Obviously, the above construction method can easily inspire us to construct the following maximally entangled quantum states

(13) $$\begin{array}{c} \begin{array}{c} |\varphi^{\prime \prime }\rangle_{12345\cdots m}=\frac{1}{\sqrt{d^{3}}}\sum_{k=0}^{d-1}{|k\rangle }_{1}\left[\sum_{j=0}^{d-1}\exp\left\{\frac{2\pi i}{d}kj\right\}{|jj\cdots j\rangle }_{23\cdots p}\right]\\ \left[\sum_{l=0}^{d-1}\exp\left\{\frac{2\pi i}{d}kl\right\}{|ll\cdots l\rangle }_{(p+1)(p+2)\cdots m}\right] \end{array} \end{array}$$

and

(14) $$\begin{array}{c} {|\varphi \rangle }_{123\cdots (2m+1)}=\frac{1}{\sqrt{d^{m+1}}}\sum_{k=0}^{d-1}{|k\rangle }_{1}\prod_{n=1}^{m}\left[\sum_{j=0}^{d-1}\exp\left\{\frac{2\pi i}{d}kj\right\}{|jj\rangle }_{2n,2n+1}\right], \end{array}$$

and so on.

The above constructions give two different classes of entangled resources. The states in Equations (11), (13) and (14) have uniform superposition coefficients and can therefore be regarded as maximally entangled resources. When such maximally entangled states are used as quantum channels, the information of an unknown input state can, in principle, be completely transmitted to the receiver through suitable joint measurements, classical communication, and the corresponding unitary correction operations. This is the basic mechanism of perfect quantum teleportation, in which both the teleportation fidelity and the success probability are equal to one [1,5,7].

By contrast, Equation (12) represents a partially entangled five-qudit state, whose coefficients $a_{kj}$ and $b_{kl}$ are generally not all equal. For a partially entangled quantum channel, after the sender performs the measurement, the state obtained by the receiver usually contains amplitude distortions associated with the channel coefficients, and such distortions generally cannot be eliminated solely by unitary correction operations [12,13]. In conventional treatments of teleportation with partially entangled channels, one possible way to overcome this difficulty is to introduce an auxiliary particle initially prepared in the state |0〉 and then perform a suitable transformation before measuring the auxiliary particle. In this procedure, the teleportation succeeds only for the desired auxiliary-particle measurement outcome, for example |0〉, whereas other outcomes, such as |1〉, correspond to failure. Thus, the whole process contains both successful and unsuccessful branches. It follows that conventional teleportation schemes based on partially entangled channels are usually probabilistic: only when specific measurement outcomes are obtained can the teleported state be recovered with unit fidelity, while the total success probability is less than one [19,20,21,22].

Based on this consideration, a natural question arises: can one eliminate the influence of amplitude distortion and realize deterministic perfect quantum teleportation through a partially entangled channel by redesigning the measurement scheme, the classical information transmission process, and the corresponding recovery operations? Motivated by this question, the following sections focus on the five-qudit partially entangled channel given in Equation (12), and investigate whether the limitations of conventional partially entangled channels can be overcome by optimizing the above transmission strategy, thereby realizing perfect quantum teleportation with fidelity equal to one and success probability equal to one.

## 3. Controlled Two-Output Teleportation of Single-Qubit States via a 3-Dimensional Partially Entangled State

In this section, we discuss the two-output teleportation of 2-dimensional single-particle states by using a 3-dimensional partially entangled state as the quantum channel under the control of the supervisor. Assume that the sender Alice, the receivers Bob and Charlie, and the controller David are four distant communicators. We set up a 3-dimensional partially entangled five-qutrit state to be used as the quantum channel among Alice, Bob, Charlie and David, which is given by

(15) $$\begin{array}{c} {|\chi \rangle }_{12345}=\frac{1}{\sqrt{3}}\sum_{k=0}^{2}{|k\rangle }_{1}\left[\sum_{j=0}^{2}a_{kj}\exp\left\{\frac{2\pi i}{3}kj\right\}{|jj\rangle }_{23}\right]\left[\sum_{l=0}^{2}b_{kl}\exp\left\{\frac{2\pi i}{3}kl\right\}{|ll\rangle }_{45}\right], \end{array}$$

where real numbers $a_{kj},b_{kl}$ (k,j,l∈{0,1,2}) satisfy the normalization conditions $\sum_{j=0}^{2}a_{kj}^{2}=1$ and $\sum_{l=0}^{2}b_{kl}^{2}=1$ for any k∈{0,1,2}. Obviously, when d=3, the state $|\varphi^{\prime }\rangle_{12345}$ shown in Equation (12) becomes the state ${|\chi \rangle }_{12345}$ shown in Equation (15). Qutrit 1 belongs to David, Alice possesses qutrits 2 and 4, while qutrits 3 and 5 belong to Bob and Charlie respectively. Without loss of generality, one can assume the Schmidt coefficients $a_{kj},b_{kl}$ (k,j,l∈{0,1,2}) satisfy $0\leq a_{k0}\leq a_{k1}\leq a_{k2}$ and $0\leq b_{k0}\leq b_{k1}\leq b_{k2}$ for any k∈{0,1,2}. Here, we set $a_{k1}=a_{k2}$ and $b_{k1}=b_{k2}$ (k=0,1,2). Suppose that Alice has two arbitrary unknown 2-dimensional single-qubit states,

(16) $$\begin{array}{c} {|\xi \rangle }_{A}=(\alpha |0\rangle +{\beta |1\rangle )}_{A}{,\;\;|\eta \rangle }_{A^{\prime }}=(x|0\rangle +{y|1\rangle )}_{A^{\prime }}, \end{array}$$

where the unknown coefficients α,β,x and *y* are complex numbers with ${\left|\alpha \right|}^{2}+{\left|\beta \right|}^{2}=1$ and ${\left|x\right|}^{2}+{\left|y\right|}^{2}=1$. She wants to respectively and perfectly teleport these states to Bob and Charlie under the control of David.

The most important step for the teleportation is Alice’s joint measurements on her particle pairs (A,2) and $\left(A^{\prime },4\right)$ separately, which project Bob’s qutrit 3, Charlie’s qutrit 5, and David’s qutrit 1 into a state dependent on Alice’s measurement results and the states ${|\xi \rangle }_{A}$ and ${|\eta \rangle }_{A^{\prime }}$. After Alice and David inform Bob and Charlie of their measurement results via the classical channels, Bob and Charlie perform corresponding operations on their respective systems to restore their target states. To teleport the states perfectly, two conditions should be satisfied as follows: (i) Alice’s measurements are all projective measurements; (ii) the collapsed states of qudits 3 or 5 are of the forms $\alpha |\tilde{0}\rangle +\beta |\tilde{1}\rangle$ or $x|\tilde{0}\rangle +y|\tilde{1}\rangle$, respectively, where $|\tilde{0}\rangle$ and $|\tilde{1}\rangle$ are two orthogonal states independent of α, β, *x* and *y*. The state of the whole initial system may be expressed as

(17) $$\begin{array}{c} \begin{array}{cc} |G\rangle & ={|\xi \rangle }_{A}⊗{|\eta \rangle }_{A^{\prime }}⊗{|\chi \rangle }_{12345}\\ & =\frac{1}{\sqrt{3}}\sum_{k=0}^{2}{|k\rangle }_{1}[a_{k0}|00\rangle \alpha |0\rangle +a_{k1}\omega^{k}|01\rangle \alpha |1\rangle +a_{k1}\omega^{2k}|02\rangle \alpha |2\rangle \\ & \;\;+a_{k0}|10\rangle \beta |0\rangle +a_{k1}\omega^{k}|11\rangle \beta |1\rangle +a_{k1}\omega^{2k}{\left|12\rangle \beta |2\rangle \right]}_{A23}\\ & \;\;⊗[b_{k0}|00\rangle x|0\rangle +b_{k1}\omega^{k}|01\rangle x|1\rangle +b_{k1}\omega^{2k}|02\rangle x|2\rangle \\ & \;\;+b_{k0}|10\rangle y|0\rangle +b_{k1}\omega^{k}|11\rangle y|1\rangle +b_{k1}\omega^{2k}{\left|12\rangle y|2\rangle \right]}_{A^{\prime }45}, \end{array} \end{array}$$

where $\omega =e^{2\pi i/3}$ is a constant.

From Equation (17), one can see that David’s particle 1 is entangled with Alice’s particles 2 and 4, Bob’s particle 3, and Charlie’s particle 5. Therefore, without David’s cooperation, Alice cannot determine the *k*-dependent measurement bases and Bob and Charlie cannot choose the correct local recovery operations. In this sense, the teleportation process is controlled by David.

**Figure 1:**
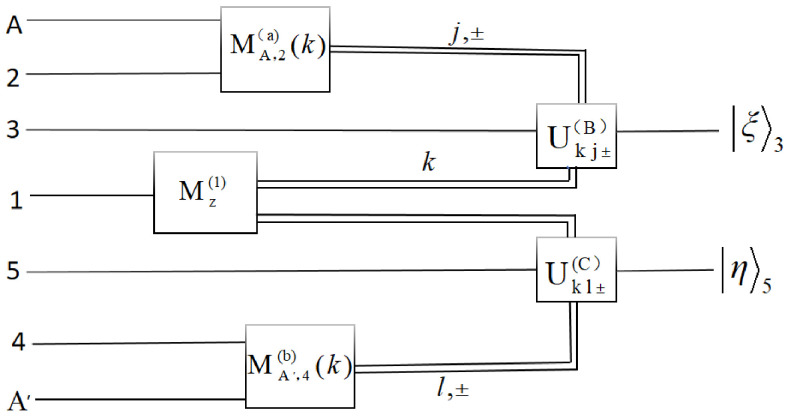
Circuit diagram of the proposed perfect controlled two-output quantum teleportation protocol for single-qubit states.

To illustrate the proposed perfect controlled two-output teleportation process via a three-dimensional partially entangled state more intuitively, the corresponding circuit diagram is shown in Figure 1.

In this diagram, the single solid lines represent quantum systems, while the double solid lines represent classical communication channels. *A* and $A^{\prime }$ denote the two unknown single-qubit states ${|\xi \rangle }_{A}$ and ${|\eta \rangle }_{A^{\prime }}$ initially held by Alice, respectively. David performs the *Z*-basis measurement $M_{Z}^{\left(1\right)}$ on particle 1 and announces the outcome *k*. According to this outcome, Alice performs the *k*-dependent joint measurements $M_{A2}^{\left(a\right)}\left(k\right)$ and $M_{A^{\prime }4}^{\left(b\right)}\left(k\right)$ on particle pairs (A,2) and $\left(A^{\prime },4\right)$, respectively. The corresponding measurement outcomes are denoted by j,± and l,±, which are sent to Bob and Charlie through classical communication channels. Then Bob and Charlie perform the corresponding local recovery operations $U_{kj\pm }^{\left(B\right)}$ and $U_{kl\pm }^{\left(C\right)}$ according to the classical information they receive. Based on the quantum circuit flow described above, the complete implementation of the teleportation scheme is presented in detail in the following three steps.

**Step 1** If the controller David is willing to cooperate with other participants, he measures his qudit 1 by using the *Z*-basis {|0〉,|1〉,|2〉}, and then publicly announces his measurement result ${|k\rangle }_{1}$ (k=0,1,2) via classical communication. Obviously, the probability of David obtaining the measurement result ${|k\rangle }_{1}$ is 1/3, and the corresponding collapsed state of the remaining particles is

(18) $$\begin{array}{c} \begin{array}{cc} |G^{\prime }\rangle & =\;[a_{k0}|00\rangle \alpha |0\rangle +a_{k1}\omega^{k}|01\rangle \alpha |1\rangle +a_{k1}\omega^{2k}|02\rangle \alpha |2\rangle \\ & \;\;+\;a_{k0}|10\rangle \beta |0\rangle +a_{k1}\omega^{k}|11\rangle \beta |1\rangle +a_{k1}\omega^{2k}{\left|12\rangle \beta |2\rangle \right]}_{A23}\\ & \;\;⊗\;[b_{k0}|00\rangle x|0\rangle +b_{k1}\omega^{k}|01\rangle x|1\rangle +b_{k1}\omega^{2k}|02\rangle x|2\rangle \\ & \;\;+\;b_{k0}|10\rangle y|0\rangle +b_{k1}\omega^{k}|11\rangle y|1\rangle +b_{k1}\omega^{2k}{\left|12\rangle y|2\rangle \right]}_{A^{\prime }45}. \end{array} \end{array}$$

**Step 2** After hearing David’s measurement information, to implement the teleportation perfectly, Alice needs to choose wisely two special orthogonal bases ${\{|\psi_{kj\pm }^{\left(a\right)}\rangle }_{A2}:j=0,1,2\}$ and ${\{|\psi_{kl\pm }^{\left(b\right)}\rangle }_{A^{\prime }4}:l=0,1,2\}$ to measure her particle pairs (A,2) and $\left(A^{\prime },4\right)$ respectively. Each of these two bases is an asymmetric basis composed of two-particle entangled states in (2×3)-dimensional Hilbert space, they are given as follows:

(19) $$\begin{array}{c} \begin{array}{cc} |\psi_{k0\pm }^{\left(a\right)}\rangle_{A2} & =\frac{1}{\sqrt{2}}[|00\rangle \;\pm \;(s_{k}|11\rangle -t_{k}{\left|02\rangle \right]}_{A2},\\ |\psi_{k1\pm }^{\left(a\right)}\rangle_{A2} & =\frac{1}{\sqrt{2}}(|01\rangle \;\pm \;{\left|12\rangle \right)}_{A2},\\ |\psi_{k2\pm }^{\left(a\right)}\rangle_{A2} & =\frac{1}{\sqrt{2}}[(s_{k}|02\rangle +t_{k}|11\rangle {)\;\pm \;|10\rangle ]}_{A2} \end{array} \end{array}$$

and

(20) $$\begin{array}{c} \begin{array}{cc} |\psi_{k0\pm }^{\left(b\right)}\rangle_{A^{\prime }4} & =\frac{1}{\sqrt{2}}[|00\rangle \pm (s_{k}^{\prime }|11\rangle -t_{k}^{\prime }{\left|02\rangle \right]}_{A^{\prime }4},\\ |\psi_{k1\pm }^{\left(b\right)}\rangle_{A^{\prime }4} & =\frac{1}{\sqrt{2}}(|01\rangle \pm {\left|12\rangle \right)}_{A^{\prime }4},\\ |\psi_{k2\pm }^{\left(b\right)}\rangle_{A^{\prime }4} & =\frac{1}{\sqrt{2}}[(s_{k}^{\prime }|02\rangle +t_{k}^{\prime }|11\rangle {)\pm |10\rangle ]}_{A^{\prime }4}, \end{array} \end{array}$$

where k∈{0,1,2}, and the real numbers $s_{k},s_{k}^{\prime },t_{k}$ and $t_{k}^{\prime }$ satisfy the following conditions:

(21) $$\begin{array}{c} \begin{array}{cc} & s_{k}=\sqrt{\frac{1}{2}\left(1+\frac{a_{k0}^{2}}{a_{k1}^{2}}\right)},\;\;t_{k}=\sqrt{\frac{1}{2}\left(1-\frac{a_{k0}^{2}}{a_{k1}^{2}}\right)},\\ & s_{k}^{\prime }=\sqrt{\frac{1}{2}\left(1+\frac{b_{k0}^{2}}{b_{k1}^{2}}\right)},\;\;t_{k}^{\prime }=\sqrt{\frac{1}{2}\left(1-\frac{b_{k0}^{2}}{b_{k1}^{2}}\right)}. \end{array} \end{array}$$

After Alice’s measurements, the (un-normalized) collapsed states of qutrits 3 and 5, corresponding to David’s outcome ${|k\rangle }_{1}$ and Alice’s measurement results $|\psi_{kj\pm }^{\left(a\right)}\rangle_{A2}$ and $|\psi_{kl\pm }^{\left(b\right)}\rangle_{A^{\prime }4}$, can be derived by

|ϕkjl±〉35=〈A′4ψkl±(b)|〈A2ψkj±(a)|G′〉,

which are given as

(22) $$\begin{array}{c} \begin{array}{cc} |\phi_{k00\pm }\rangle_{35} & =\frac{1}{2}{[\alpha {(a_{k0}|0\rangle }_{3}\mp a_{k1}t_{k}\omega^{2k}|2\rangle }_{3})\;\pm \;\beta a_{k1}s_{k}\omega^{k}{|1\rangle }_{3}]\\ \;\; & ⊗{[x{(b_{k0}|0\rangle }_{5}\mp b_{k1}t_{k}^{\prime }\omega^{2k}|2\rangle }_{5})\;\pm \;yb_{k1}s_{k}^{\prime }\omega^{k}{|1\rangle }_{5}],\\ |\phi_{k01\pm }\rangle_{35} & =\frac{1}{2}{[\alpha {(a_{k0}|0\rangle }_{3}\mp a_{k1}t_{k}\omega^{2k}|2\rangle }_{3})\;\pm \;\beta a_{k1}s_{k}\omega^{k}{|1\rangle }_{3}]\\ \;\; & ⊗{[xb_{k1}\omega^{k}|1\rangle }_{5}\;\pm \;yb_{k1}\omega^{2k}{|2\rangle }_{5}],\\ |\phi_{k02\pm }\rangle_{35} & =\frac{1}{2}{[\alpha {(a_{k0}|0\rangle }_{3}\mp a_{k1}t_{k}\omega^{2k}|2\rangle }_{3})\;\pm \;\beta a_{k1}s_{k}\omega^{k}{|1\rangle }_{3}]\\ \;\; & ⊗{[xb_{k1}s_{k}^{\prime }\omega^{2k}|2\rangle }_{5}+y{(b_{k1}t_{k}^{\prime }\omega^{k}|1\rangle }_{5}\;\pm \;b_{k0}{|0\rangle }_{5})],\\ |\phi_{k10\pm }\rangle_{35} & =\frac{1}{2}{[\alpha a_{k1}\omega^{k}|1\rangle }_{3}\;\pm \;\beta a_{k1}\omega^{2k}{|2\rangle }_{3}]\\ \;\; & ⊗{[x{(b_{k0}|0\rangle }_{5}\mp b_{k1}t_{k}^{\prime }\omega^{2k}|2\rangle }_{5})\;\pm \;yb_{k1}s_{k}^{\prime }\omega^{k}{|1\rangle }_{5}],\\ |\phi_{k11\pm }\rangle_{35} & =\frac{1}{2}{[\alpha a_{k1}\omega^{k}|1\rangle }_{3}\;\pm \;\beta a_{k1}\omega^{2k}{|2\rangle }_{3}]\\ \;\; & ⊗{[xb_{k1}\omega^{k}|1\rangle }_{5}\;\pm \;yb_{k1}\omega^{2k}{|2\rangle }_{5}],\\ |\phi_{k12\pm }\rangle_{35} & =\frac{1}{2}{[\alpha a_{k1}\omega^{k}|1\rangle }_{3}\;\pm \;\beta a_{k1}\omega^{2k}{|2\rangle }_{3}]\\ \;\; & ⊗{[xb_{k1}s_{k}^{\prime }\omega^{2k}|2\rangle }_{5}+y{(b_{k1}t_{k}^{\prime }\omega^{k}|1\rangle }_{5}\;\pm \;b_{k0}{|0\rangle }_{5})],\\ |\phi_{k20\pm }\rangle_{35} & =\frac{1}{2}{[\alpha a_{k1}s_{k}\omega^{2k}|2\rangle }_{3}+\beta {(a_{k1}t_{k}\omega^{k}|1\rangle }_{3}\;\pm \;a_{k0}{|0\rangle }_{3})]\\ \;\; & ⊗{[x{(b_{k0}|0\rangle }_{5}\mp b_{k1}t_{k}^{\prime }\omega^{2k}|2\rangle }_{5})\;\pm \;yb_{k1}s_{k}^{\prime }\omega^{k}{|1\rangle }_{5}],\\ |\phi_{k21\pm }\rangle_{35} & =\frac{1}{2}{[\alpha a_{k1}s_{k}\omega^{2k}|2\rangle }_{3}+\beta {(a_{k1}t_{k}\omega^{k}|1\rangle }_{3}\;\pm \;a_{k0}{|0\rangle }_{3})]\\ \;\; & ⊗{[xb_{k1}\omega^{k}|1\rangle }_{5}\;\pm \;yb_{k1}\omega^{2k}{|2\rangle }_{5}],\\ |\phi_{k22\pm }\rangle_{35} & =\frac{1}{2}{[\alpha a_{k1}s_{k}\omega^{2k}|2\rangle }_{3}+\beta {(a_{k1}t_{k}\omega^{k}|1\rangle }_{3}\;\pm \;a_{k0}{|0\rangle }_{3})]\\ \;\; & ⊗{[xb_{k1}s_{k}^{\prime }\omega^{2k}|2\rangle }_{5}+y{(b_{k1}t_{k}^{\prime }\omega^{k}|1\rangle }_{5}\;\pm \;b_{k0}{|0\rangle }_{5})] \end{array} \end{array}$$

for all k∈{0,1,2}. Then Alice informs Bob and Charlie of her measurement results $|\psi_{kj\pm }^{\left(a\right)}\rangle_{A2}$ and $|\psi_{kl\pm }^{\left(b\right)}\rangle_{A^{\prime }4}$ respectively through the classical channels.

It is worth mentioning that the ingenious construction of the asymmetric measurement bases shown in Equations (19)–(21) comes from the consideration of Equation (17) and the application of condition (ii) and $s_{k}^{2}+t_{k}^{2}=1$ and ${s^{\prime }}_{k}^{2}+{t^{\prime }}_{k}^{2}=1$.

By calculation, the probabilities of Alice’s outcomes $|\psi_{kj\pm }^{\left(a\right)}\rangle_{A2}$ and $|\psi_{kl\pm }^{\left(b\right)}\rangle_{A^{\prime }4}$ appearing are given by the overlap Pkjl±=〈35ϕkjl±|ϕkjl±〉35 as

(23) $$\begin{array}{c} \begin{array}{cc} P_{k00\pm } & =P_{k02\pm }=P_{k20\pm }=P_{k22\pm }=\frac{1}{16}\left(a_{k0}^{2}+a_{k1}^{2}\right)\left(b_{k0}^{2}+b_{k1}^{2}\right),\\ P_{k01\pm } & =P_{k21\pm }=\frac{1}{8}\left(a_{k0}^{2}+a_{k1}^{2}\right)b_{k1}^{2},\\ P_{k10\pm } & =P_{k12\pm }=\frac{1}{8}a_{k1}^{2}\left(b_{k0}^{2}+b_{k1}^{2}\right),\\ P_{k11\pm } & =\frac{1}{4}a_{k1}^{2}b_{k1}^{2}. \end{array} \end{array}$$

**Step 3** According to the measurement information from David and Alice, Bob and Charlie perform appropriate local operations to reconstruct their respective target states. For example, if David’s measurement outcome is ${|k\rangle }_{1}$ and Alice’s joint measurement results are $|\psi_{k0+}^{\left(a\right)}\rangle_{A2}$ and $|\psi_{k2-}^{\left(b\right)}\rangle_{A^{\prime }4}$, then the state of qutrits 3 and 5 collapses into

(24) $$\begin{array}{c} \begin{array}{cc} |\phi_{k02+-}\rangle_{35} & =\frac{1}{2}{[\alpha {(a_{k0}|0\rangle }_{3}-a_{k1}t_{k}\omega^{2k}|2\rangle }_{3})+\beta a_{k1}s_{k}\omega^{k}{|1\rangle }_{3}]\\ & \;\;⊗\;{[xb_{k1}s_{k}^{\prime }\omega^{2k}|2\rangle }_{5}+y{(b_{k1}t_{k}^{\prime }\omega^{k}|1\rangle }_{5}-b_{k0}{|0\rangle }_{5})]. \end{array} \end{array}$$

Bob needs to performs the local operation

(25) $$\begin{array}{c} U_{k0+}^{\left(B\right)}=s_{k}\left|0\rangle \langle 0\right|-\frac{a_{k0}}{a_{k1}}\omega^{-2k}\left|2\rangle \langle 2\right|+\frac{a_{k0}}{a_{k1}s_{k}}\omega^{-k}\left|1\rangle \langle 1\right| \end{array}$$

on his qutrit 3, and Charlie executes the local operation

(26) $$\begin{array}{c} U_{k2-}^{\left(C\right)}=\frac{b_{k0}}{b_{k1}s_{k}^{\prime }}\omega^{-2k}\left|2\rangle \langle 2\right|+\frac{b_{k0}}{b_{k1}}\omega^{-k}\left|1\rangle \langle 1\right|-s_{k}^{\prime }\left|0\rangle \langle 0\right| \end{array}$$

on his qutrit 5. Then the state $|\phi_{k02+-}\rangle_{35}$ becomes

(27) $$\begin{array}{c} \begin{array}{cc} \left(U_{k0+}^{\left(B\right)}⊗U_{k2-}^{\left(C\right)}\right){|\phi_{k02+-}\rangle }_{35} & =\frac{1}{2}a_{k0}b_{k0}[\alpha (s_{k}|0\rangle +t_{k}|2\rangle {)}_{3}\\ \;\; & +{\beta |1\rangle }_{3}{]⊗[x|2\rangle }_{5}+y(s_{k}^{\prime }|0\rangle +t_{k}^{\prime }{\left|1\rangle \right)}_{5}]. \end{array} \end{array}$$

Let $|\tilde{0}\rangle_{3}=(s_{k}|0\rangle +t_{k}{\left|2\rangle \right)}_{3}$ and $|\tilde{1}\rangle_{5}=(s_{k}^{\prime }|0\rangle +t_{k}^{\prime }{\left|1\rangle \right)}_{5}$. Obviously, each of the two product vectors on the right-hand side of Equation (27) satisfies the condition (ii), so Bob and Charlie have successfully reconstructed their respective target states, completing the teleportation.

For a fixed value *k*, similar analysis methods can be used to obtain corresponding conclusions about Alice’s other measurement results. The measurement results of Alice, the local operations of Bob and Charlie, and their reconstructed target states are summarized in Table 1, where $U_{k0\pm }^{\left(B\right)}=s_{k}\left|0\rangle \langle 0\right|\mp \omega^{-2k}a_{k0}/a_{k1}\left|2\rangle \langle 2\right|\pm \omega^{-k}a_{k0}/\left(a_{k1}s_{k}\right)\left|1\rangle \langle 1\right|$, $U_{k0\pm }^{\left(C\right)}=s_{k}^{\prime }\left|0\rangle \langle 0\right|\mp \omega^{-2k}b_{k0}/b_{k1}\left|2\rangle \langle 2\right|\pm \omega^{-k}b_{k0}/\left(b_{k1}s_{k}^{\prime }\right)\left|1\rangle \langle 1\right|$, $U_{k1\pm }=\left|0\rangle \langle 0\right|+\omega^{-k}\left|1\rangle \langle 1\right|\pm \omega^{-2k}\left|2\rangle \langle 2\right|$, $U_{k2\pm }^{\left(B\right)}=\omega^{-2k}a_{k0}/\left(a_{k1}s_{k}\right)\left|2\rangle \langle 2\right|+\omega^{-k}a_{k0}/a_{k1}\left|1\rangle \langle 1\right|\pm s_{k}\left|0\rangle \langle 0\right|$ and $U_{k2\pm }^{\left(C\right)}=\omega^{-2k}$$b_{k0}/\left(b_{k1}s_{k}^{\prime }\right)\left|2\rangle \langle 2\right|+\omega^{-k}b_{k0}/b_{k1}\left|1\rangle \langle 1\right|\pm s_{k}^{\prime }\left|0\rangle \langle 0\right|$.

Now, let’s calculate the overall success probability of the proposed scheme. For the fixed values k,j and *l*, $\sum_{+,-}P_{kjl\pm }$ means the sum of the probabilities of Alice’s four measurements $|\psi_{kj+}^{\left(a\right)}\rangle_{A2}{|\psi_{kl+}^{\left(b\right)}\rangle }_{A^{\prime }4}$, $|\psi_{kj+}^{\left(a\right)}\rangle_{A2}{|\psi_{kl-}^{\left(b\right)}\rangle }_{A^{\prime }4}$, $|\psi_{kj-}^{\left(a\right)}\rangle_{A2}{|\psi_{kl+}^{\left(b\right)}\rangle }_{A^{\prime }4}$ and $|\psi_{kj-}^{\left(a\right)}\rangle_{A2}{|\psi_{kl-}^{\left(b\right)}\rangle }_{A^{\prime }4}$ occurring, and the probability of any of them appearing is the same value $P_{kjl\pm }$. From step 3, it is known that when *k* is fixed, quantum teleportation can succeed for any measurement result of Alice. This means that the success probability of quantum teleportation under condition *k* is equal to the sum of all probabilities for Alice’s measurement results occurring under condition *k*, i.e.,

(28) $$\begin{array}{c} \begin{array}{cc} \sum_{j,l,+,-}P_{kjl\pm } & =4\left(P_{k00\pm }+P_{k0l\pm }+P_{k02\pm }\right)+4\left(P_{k10\pm }+P_{k1l\pm }+P_{k12\pm }\right)\\ \;\; & +\;4(P_{k20\pm }+P_{k2l\pm }+P_{k22\pm })\\ & =\left[\frac{1}{2}\left(a_{k0}^{2}+a_{k1}^{2}\right)\left(b_{k0}^{2}+b_{k1}^{2}\right)+\frac{1}{2}\left(a_{k0}^{2}+a_{k1}^{2}\right)b_{k1}^{2}\right]+\left[a_{k1}^{2}\left(b_{k0}^{2}+b_{k1}^{2}\right)+a_{k1}^{2}b_{k1}^{2}\right]\\ & \;+\;[\frac{1}{2}\left(a_{k0}^{2}+a_{k1}^{2}\right)\left(b_{k0}^{2}+b_{k1}^{2}\right)+\frac{1}{2}\left(a_{k0}^{2}+a_{k1}^{2}\right)b_{k1}^{2}]\\ & =\left(a_{k0}^{2}+2a_{k1}^{2}\right)\left(b_{k0}^{2}+2b_{k1}^{2}\right)=1 \end{array} \end{array}$$

because of $a_{k0}^{2}+2a_{k1}^{2}=b_{k0}^{2}+2b_{k1}^{2}=1$. That is, for any k∈{0,1,2}, the two-output teleportation is always achieved with a probability of 1. Note that the probability of David obtaining measurement result ${|k\rangle }_{1}$ is 1/3, and there are a total of 3 possible measurement results for David. Therefore, the overall success probability of our scheme here is $(\frac{1}{3}\times 1)\times 3=1$. Based on the conclusion of step 3, we have demonstrated that quantum teleportation of single-particle states through three-dimensional partially entangled quantum channels can be perfectly achieved.

## 4. Controlled Two-Output Teleportation of Single-Qubit States Through a *D*-Dimensional Partially Entangled State

Now we extend the scheme in Section 3 to the general scheme for teleporting two unknown single-qubit states via the partially entangled five-qudit state shown in Equation (12), where the real Schmidt coefficients $0\leq a_{0}\leq a_{1}\leq a_{2}\leq \cdots a_{d-2}=a_{d-1}$ and $0\leq b_{0}\leq b_{1}\leq b_{2}\leq \cdots b_{d-2}=b_{d-1}$. Our only requirement on the quantum channel is that the two largest Schmidt coefficients are equal, and the other assumptions in Section 3 remain unchanged. Since the present scheme is a *d*-dimensional generalization of the three-dimensional scheme in Section 3, its circuit structure and classical communication pattern are similar to those shown in Figure 1. The main difference is that the channel dimension, Alice’s measurement bases and the receivers’ recovery operations are generalized from the three-dimensional case to the *d*-dimensional case. Therefore, to avoid repetition, the corresponding circuit diagram is not redrawn in this section. The initial total state of the system in the teleportation can be written as

(29) $$\begin{array}{c} \begin{array}{cc} |\Xi \rangle & ={|\xi \rangle }_{A}⊗{|\eta \rangle }_{A^{\prime }}⊗{|\varphi^{\prime }\rangle }_{12345}\\ & =\frac{1}{\sqrt{d}}\sum_{k=0}^{d-1}{|k\rangle }_{1}\left[\sum_{j=0}^{d-1}a_{kj}\exp\left\{\frac{2\pi i}{d}kj\right\}{(|0j\rangle }_{A2}{\alpha |j\rangle }_{3}+{|1j\rangle }_{A2}{\beta |j\rangle }_{3})\right]\\ & \;⊗\left[\sum_{l=0}^{d-1}b_{kl}\exp\left\{\frac{2\pi i}{d}kl\right\}{(|0l\rangle }_{A^{\prime }4}{x|l\rangle }_{5}+{|1l\rangle }_{A^{\prime }4}{y|l\rangle }_{5})\right]. \end{array} \end{array}$$

From Equation (29), it can be inferred that David’s cooperation determines whether the teleportation process can be activated. If David is willing to collaborate with the other communication participants, he executes a single-qudit projection measurement on his qudit 1 in the computational basis {|0〉,|1〉,⋯,|d−1〉}, and then publicly discloses his measurement result ${|k\rangle }_{1}$ (k=0,1,⋯,d−1) to the participants through classical communication channels. Obviously, the probability of David obtaining each measurement result ${|k\rangle }_{1}$ is 1/d, and the corresponding collapsed state is

(30) $$\begin{array}{c} \begin{array}{cc} |\Xi^{\prime }\rangle & =\left[\sum_{j=0}^{d-1}a_{kj}\exp\left\{\frac{2\pi i}{d}kj\right\}{(|0j\rangle }_{A2}{\alpha |j\rangle }_{3}+{|1j\rangle }_{A2}{\beta |j\rangle }_{3})\right]\\ & \;⊗\left[\sum_{l=0}^{d-1}b_{kl}\exp\left\{\frac{2\pi i}{d}kl\right\}{(|0l\rangle }_{A^{\prime }4}{x|l\rangle }_{5}+{|1l\rangle }_{A^{\prime }4}{y|l\rangle }_{5})\right]. \end{array} \end{array}$$

The sender Alice selects the two special measurement bases ${\{|\Psi_{kj\pm }^{\left(a\right)}\rangle }_{A2}:j=0,1,\cdots ,d-1\}$ and ${\{|\Psi_{kl\pm }^{\left(b\right)}\rangle }_{A^{\prime }4}:l=0,1,\cdots ,d-1\}$ to measure her pairs (A,2) and $\left(A^{\prime },4\right)$ respectively. Each group of bases is an asymmetric basis composed of vectors in a (2×d)-dimensional Hilbert space, they are given as follows:

(31) $$\begin{array}{c} \begin{array}{cc} & |\Psi_{kj\pm }^{\left(a\right)}\rangle_{A2}=|0,j\rangle \pm s_{j}|1,j+1\rangle \;\mp \;t_{j}(\prod_{m=j+1}^{d-3}s_{m}|0,d-1\rangle +\sum_{n=j+1}^{d-3}\prod_{m=j+1}^{n-1}s_{m}t_{n}|1,n+1\rangle ),\\ & |\Psi_{k(d-1)\pm }^{\left(a\right)}\rangle_{A2}=(\prod_{m=0}^{d-3}s_{m}|0,d-1\rangle +\sum_{n=0}^{d-3}\prod_{m=0}^{n-1}s_{m}t_{n}|1,n+1\rangle )\pm |10\rangle , \end{array} \end{array}$$

and

(32) $$\begin{array}{c} \begin{array}{cc} & |\Psi_{kl\pm }^{\left(b\right)}\rangle_{A^{\prime }4}=|0,l\rangle \pm s_{l}^{\prime }|1,l+1\rangle \;\mp \;t_{l}^{\prime }(\prod_{m=l+1}^{d-3}s_{m}^{\prime }|0,d-1\rangle +\sum_{n=l+1}^{d-3}\prod_{m=l+1}^{n-1}s_{m}^{\prime }t_{n}^{\prime }|1,n+1\rangle ),\\ & |\Psi_{k(d-1)\pm }^{\left(b\right)}\rangle_{A^{\prime }4}=(\prod_{m=0}^{d-3}s_{m}^{\prime }|0,d-1\rangle +\sum_{n=0}^{d-3}\prod_{m=0}^{n-1}s_{m}^{\prime }t_{n}^{\prime }|1,n+1\rangle )\pm |10\rangle , \end{array} \end{array}$$

with

(33) $$\begin{array}{c} \begin{array}{cc} & s_{r}=\sqrt{\frac{1}{2}\left(1+\frac{a_{kr}^{2}}{a_{k(r+1)}^{2}}\right)},\;\;t_{r}=\sqrt{\frac{1}{2}\left(1-\frac{a_{kr}^{2}}{a_{k(r+1)}^{2}}\right)},\\ & s_{r}^{\prime }=\sqrt{\frac{1}{2}\left(1+\frac{b_{kr}^{2}}{b_{k(r+1)}^{2}}\right)},\;\;t_{r}^{\prime }=\sqrt{\frac{1}{2}\left(1-\frac{b_{kr}^{2}}{b_{k(r+1)}^{2}}\right)}, \end{array} \end{array}$$

where we omit thier normalization coefficients $1/\sqrt{2}$ and subscript identifying Alice’s subsystems.

After Alice’s measurements, the (unnormalized) collapsed states of qudits 3 and 5, corresponding to measurement bases shown in Equations (31) and (32), are given by

(34) $$\begin{array}{c} \begin{array}{cc} |\lambda_{kjl\pm }\rangle_{35} & =[\alpha {(a_{kj}\zeta^{kj}|j\rangle }_{3}\mp t_{j}\prod_{m=j+1}^{d-3}s_{m}a_{k(d-1)}\zeta^{k(d-1)}{|d-1\rangle }_{3})\\ & \;\;\pm \;\beta {(s_{j}a_{k(j+1)}\zeta^{k(j+1)}|j+1\rangle }_{3}-t_{j}\sum_{n=j+1}^{d-3}\prod_{m=j+1}^{n-1}s_{m}t_{n}a_{k(n+1)}\zeta^{k(n+1)}{|n+1\rangle }_{3})]\\ & \;\;⊗\;[x{(b_{kl}\zeta^{kl}|l\rangle }_{5}\mp t_{l}^{\prime }\prod_{m=l+1}^{d-3}s_{m}^{\prime }b_{k(d-1)}\zeta^{k(d-1)}|{d-1\rangle }_{5})\\ & \;\;\pm \;y{(s_{l}^{\prime }b_{k(l+1)}\zeta^{k(l+1)}|l+1\rangle }_{5}-t_{l}^{\prime }\sum_{n=l+1}^{d-3}\prod_{m=l+1}^{n-1}s_{m}^{\prime }t_{n}^{\prime }b_{k(n+1)}\zeta^{k(n+1)}{|n+1\rangle }_{5})], \end{array} \end{array}$$

and

(35) $$\begin{array}{c} \begin{array}{cc} |\lambda_{k(d-1)(d-1)\pm }\rangle_{35} & =[\alpha (\prod_{m=0}^{d-3}s_{m}a_{k(d-1)}\zeta^{k(d-1)}|{d-1\rangle }_{3})\\ \;\; & +\;\beta (\sum_{n=0}^{d-3}\prod_{m=0}^{n-1}s_{m}t_{n}a_{k(n+1)}\zeta^{k(n+1)}{|n+1\rangle }_{3}\pm a_{k0}{|0\rangle }_{3})]\\ & \;⊗\;[x(\prod_{m=0}^{d-3}s_{m}^{\prime }b_{k(d-1)}\zeta^{k(d-1)}{|d-1\rangle }_{5})\\ \;\; & +\;y(\sum_{n=0}^{d-3}\prod_{m=0}^{n-1}s_{m}^{\prime }t_{n}^{\prime }b_{k(n+1)}\zeta^{k(n+1)}{|n+1\rangle }_{5}\pm b_{k0}{|0\rangle }_{5})], \end{array} \end{array}$$

where j,l=0,1,…,d−2 and $\zeta =e^{\frac{2\pi i}{d}}$. Then, via classical channels, Alice informs Bob of the measurement outcome obtained on particle pair (A,2), namely $|\Psi_{kj\pm }^{\left(a\right)}\rangle_{A2}$ or $|\Psi_{k(d-1)\pm }^{\left(a\right)}\rangle_{A2}$, and informs Charlie of the measurement outcome obtained on particle pair $\left(A^{\prime },4\right)$, namely $|\Psi_{kl\pm }^{\left(b\right)}\rangle_{A^{\prime }4}$ or $|\Psi_{k(d-1)\pm }^{\left(b\right)}\rangle_{A^{\prime }4}$.

According to the classical information of the measurement results from David and Alice, Bob performs a unitary operation $U_{B}=\zeta^{-kj}{|j\rangle }_{3}\langle j|+\zeta^{-k(d-1)}{|d-1\rangle }_{3}\langle d-1|$$\;+\;\;\zeta^{-k(j+1)}{|j+1\rangle }_{3}\langle j+1|\;+\;\zeta^{-k(n+1)}{|n+1\rangle }_{3}\langle n+1|$ or $U_{B}=\zeta^{-k(d-1)}{|d-1\rangle }_{3}\langle d-1|\;+\;\zeta^{-k(n+1)}{|n+1\rangle }_{3}\langle n+1|$ on his qudit 3. Meanwhile, Charlie also carries out a unitary operation $U_{C}=\zeta^{-kl}{|l\rangle }_{5}\langle l|\;+\;\zeta^{-k(d-1)}{|d-1\rangle }_{5}\langle d-1|\;+\;\zeta^{-k(l+1)}{|l+1\rangle }_{5}\langle l+1|\;+\;\zeta^{-k(n+1)}{|n+1\rangle }_{5}\langle n+1|$ or $U_{C}=\zeta^{-k(d-1)}{|d-1\rangle }_{5}\langle d-1|\;+\;\zeta^{-k(n+1)}{|n+1\rangle }_{5}\langle n+1|$ on his qudit 5. Then, the states shown in Equations (34) and (35) transform into

(36) $$\begin{array}{c} \begin{array}{cc} |\lambda_{kjl\pm }^{\prime }\rangle_{35} & ={[\alpha (a_{kj}|j\rangle }_{3}\mp t_{j}\prod_{m=j+1}^{d-3}s_{m}a_{k(d-1)}{|d-1\rangle }_{3})\\ \;\; & \pm \;\beta {(s_{j}a_{k(j+1)}|j+1\rangle }_{3}-t_{j}\sum_{n=j+1}^{d-3}\prod_{m=j+1}^{n-1}s_{m}t_{n}a_{k(n+1)}{|n+1\rangle }_{3})]\\ & \;\;⊗\;[x{(b_{kl}|l\rangle }_{5}\mp t_{l}^{\prime }\prod_{m=l+1}^{d-3}s_{m}^{\prime }b_{k(d-1)}{|d-1\rangle }_{5})\pm y{(s_{l}^{\prime }b_{k(l+1)}|l+1\rangle }_{5}\\ \;\; & -t_{l}^{\prime }\sum_{n=l+1}^{d-3}\prod_{m=l+1}^{n-1}s_{m}^{\prime }t_{n}^{\prime }b_{k(n+1)}{|n+1\rangle }_{5})], \end{array} \end{array}$$

and

(37) $$\begin{array}{c} \begin{array}{cc} |\lambda_{k(d-1)(d-1)\pm }^{\prime }\rangle_{35} & =\left[\alpha (\prod_{m=0}^{d-3}s_{m}a_{k(d-1)}{|d-1\rangle }_{3}\right)+\beta (\sum_{n=0}^{d-3}\prod_{m=0}^{n-1}s_{m}t_{n}a_{k(n+1)}{|n+1\rangle }_{3}\pm a_{k0}{|0\rangle }_{3})]\\ & \;\;⊗\left[x(\prod_{m=0}^{d-3}s_{m}^{\prime }b_{k(d-1)}{|d-1\rangle }_{5}\right)+y(\sum_{n=0}^{d-3}\prod_{m=0}^{n-1}s_{m}^{\prime }t_{n}^{\prime }b_{k(n+1)}{|n+1\rangle }_{5}\pm b_{k0}{|0\rangle }_{5})]. \end{array} \end{array}$$

By calculation, the corresponding probabilities of Alice’s outcomes $|\Psi_{kj\pm }^{\left(a\right)}\rangle_{A2}$, $|\Psi_{k(d-1)\pm }^{\left(a\right)}\rangle_{A2}$, $|\Psi_{kl\pm }^{\left(b\right)}\rangle_{A^{\prime }4}$ and $|\Psi_{k(d-1)\pm }^{\left(b\right)}\rangle_{A^{\prime }4}$ are respectively

(38) $$\begin{array}{c} \begin{array}{cc} P_{kj\pm } & =\frac{1}{2}\left(a_{kj}^{2}+t_{j}^{2}\prod_{m=j+1}^{d-3}s_{m}^{2}a_{k(d-1)}^{2}\right),\;\;P_{k(d-1)\pm }=\frac{1}{2}\prod_{m=0}^{d-3}s_{m}^{2}a_{k(d-1)}^{2},\\ P_{kl\pm }^{\prime } & =\frac{1}{2}\left(b_{kl}^{2}+t_{l}^{\prime 2}\prod_{m=l+1}^{d-3}s_{m}^{\prime 2}b_{k(d-1)}^{2}\right),\;\;P_{k(d-1)\pm }^{\prime }=\frac{1}{2}\prod_{m=0}^{d-3}s_{m}^{\prime 2}b_{k(d-1)}^{2}. \end{array} \end{array}$$

Direct calculation shows the probability amplitudes in the above results satisfy

(39) $$\begin{array}{c} \begin{array}{cc} & a_{kj}^{2}+t_{j}^{2}\prod_{m=j+1}^{d-3}s_{m}^{2}a_{k(d-1)}^{2}=s_{j}^{2}a_{k(j+1)}^{2}+t_{j}^{2}\sum_{n=j+1}^{d-3}\prod_{m=j+1}^{n-1}s_{m}^{2}t_{n}^{2}a_{k(n+1)}^{2},\\ & \prod_{m=0}^{d-3}s_{m}^{2}a_{k(d-1)}^{2}=\sum_{n=0}^{d-3}\prod_{m=0}^{n-1}s_{m}^{2}t_{n}^{2}a_{k(n+1)}^{2}+a_{k0}^{2},\\ & b_{kl}^{2}+t_{l}^{\prime 2}\prod_{m=l+1}^{d-3}s_{m}^{\prime 2}b_{k(d-1)}^{2}=s_{l}^{\prime 2}b_{k(l+1)}^{2}+t_{l}^{\prime 2}\sum_{n=l+1}^{d-3}\prod_{m=l+1}^{n-1}s_{m}^{\prime 2}t_{n}^{\prime 2}b_{k(n+1)}^{2},\\ & \prod_{m=0}^{d-3}s_{m}^{\prime 2}b_{k(d-1)}^{2}=\sum_{n=0}^{d-3}\prod_{m=0}^{n-1}s_{m}^{\prime 2}t_{n}^{\prime 2}b_{k(n+1)}^{2}+b_{k0}^{2}. \end{array} \end{array}$$

Therefore, the quantum states shown in Equations (36) and (37) are of the form $(\alpha |\tilde{0}\rangle +\beta |\tilde{1}\rangle )⊗(x|\hat{0}\rangle +y|\hat{1}\rangle )$. Consequently, based on the classical information of the measurement results from David and Alice, the receivers Bob and Charlie can respectively and perfectly transform their respective states into ${|\xi \rangle }_{3}$ and ${|\eta \rangle }_{5}$ by appropriate local unitary operations.

## 5. Controlled Multi-Output Teleportation of Single-Qubit States Through a High-Dimensional Partially Entangled State

In this section, we extend the above two-output quantum teleportation schemes to the case of any multiple outputs. For clarity of thought and concise expression, we may consider teleportation through three-dimensional partially entangled state as the quantum channel. Suppose that sender Alice, *M* receivers Bob_1_, Bob_2_, …, Bob_*M*_, and supervisor Charlie are legitimate participants in spatial separation, and they share in advance the following partially entangled state in a three-dimensional Hilbert space:

(40) $$\begin{array}{c} {|\varphi \rangle }_{123\cdots (2M+1)}=\frac{1}{\sqrt{3}}\sum_{k=0}^{2}{|k\rangle }_{1}\prod_{n=1}^{M}\left[\sum_{j=0}^{2}b_{kn_{j}}\exp\left\{\frac{2\pi i}{3}kj\right\}{|jj\rangle }_{2n,2n+1}\right] \end{array}$$

with $\sum_{j=0}^{2}{\left|b_{kn_{j}}\right|}^{2}=1$ for any k∈{0,1,2} and n∈{1,2,⋯,M}, which is derived from a more general scenario of the state shown in Equation (14) under d=3. Qutrit 1 belongs to Charlie, Alice possesses qutrits 2,4,⋯,2M, while qutrits 3,5,⋯,2M+1 belong to Bob_1_, Bob_2_, ⋯, Bob_*M*_ respectively. Without loss of generality, we can assume the Schmidt coefficients $b_{kn_{j}}$ (k,j∈{0,1,2} and n∈{1,2,⋯,M}) are real numbers and $0\leq b_{kn_{0}}\leq b_{kn_{1}}=b_{kn_{2}}$. Suppose that sender Alice intends to teleport the states $|\xi_{1}\rangle_{A_{1}},{|\xi_{2}\rangle }_{A_{2}},\cdots$, and $|\xi_{M}\rangle_{A_{M}}$ to Bob_1_, Bob_2_, ⋯, and Bob_*M*_ respectively, under the control of the supervisor Charlie. These states are of the form

(41) $$\begin{array}{c} |\xi_{n}\rangle_{A_{n}}=\alpha_{n}{|0\rangle }_{A_{n}}+\beta_{n}{|1\rangle }_{A_{n}} \end{array}$$

with $|\alpha_{n}{|}^{2}+{\left|\beta_{n}\right|}^{2}=1$ for any n∈{1,2,⋯,M}.

The total state of the initial system is written as

(42) $$\begin{array}{c} \begin{array}{cc} |\Omega \rangle & =\prod_{n=1}^{M}|\xi_{n}\rangle_{A_{n}}⊗{|\varphi \rangle }_{123\cdots (2M+1)}\\ & =\frac{1}{\sqrt{3}}\sum_{k=0}^{2}{|k\rangle }_{1}\prod_{n=1}^{M}{(b_{kn_{0}}|00\rangle }_{A_{n},2n}\alpha_{n}{|0\rangle }_{2n+1}+b_{kn_{1}}\omega^{k}{|01\rangle }_{A_{n},2n}\alpha_{n}{|1\rangle }_{2n+1}\\ & \;\;+b_{kn_{1}}\omega^{2k}{|02\rangle }_{A_{n},2n}\alpha_{n}{|2\rangle }_{2n+1}+b_{kn_{0}}{|10\rangle }_{A_{n},2n}\beta_{n}{|0\rangle }_{2n+1}\\ & \;\;+b_{kn_{1}}\omega^{k}{|11\rangle }_{A_{n},2n}\beta_{n}{|1\rangle }_{2n+1}+b_{kn_{1}}\omega^{2k}{|12\rangle }_{A_{n},2n}\beta_{n}{|2\rangle }_{2n+1}), \end{array} \end{array}$$

where ω=exp(2πi/3).

**Figure 2:**
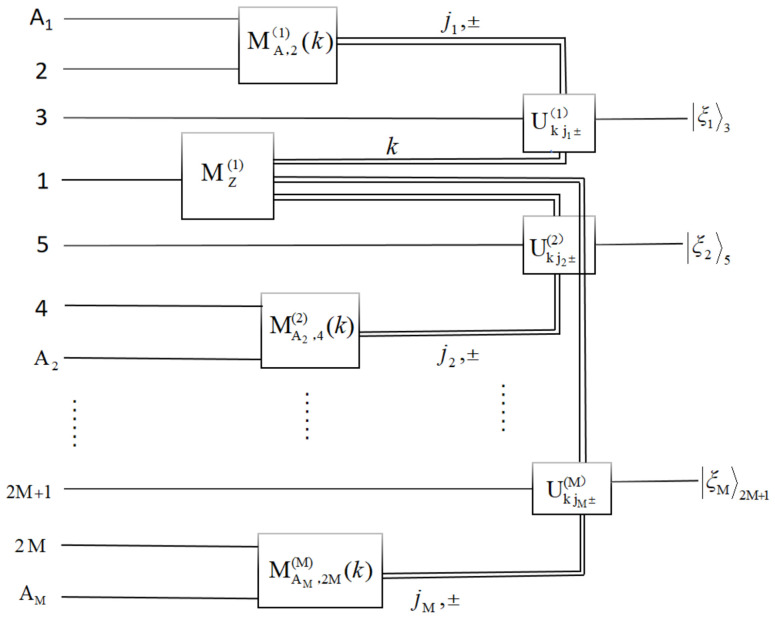
Quantum circuit for controlled multi-output teleportation of single-qubit states through a three-dimensional partially entangled state. Here, $M_{A_{n},2n}^{\left(n\right)}\left(k\right)$ denotes the *k*-dependent asymmetric joint measurement performed on the particle pair $\left(A_{n},2n\right)$, $M_{Z}^{\left(1\right)}$ denotes the measurement on particle 1 in the *Z* basis, and $U_{kj_{n}\pm }^{\left(n\right)}$ denotes the corresponding local recovery operation performed by Bob_*n*_ on particle 2n+1 to reconstruct the target state. The symbols *k* and $j_{n},\;\pm$ represent the corresponding measurement outcomes transmitted through classical communication channels, where n=1,2,…,M. The single solid lines and double solid lines represent quantum channels and classical communication channels, respectively.

To provide a more intuitive illustration of the controlled multi-output teleportation of single-qubit states through a three-dimensional partially entangled state, we present the corresponding quantum circuit in Figure 2. The whole multi-output teleportation protocol can then be implemented through the following three steps.

**Step 1** If the supervisor Charlie is willing to collaborate with his colleagues, he should carry out a single-qutrit projective measurement on his qutrit 1 in the *Z*-basis {|0〉,|1〉,|2〉}, and then he discloses the classical information of his measurement result ${|k\rangle }_{1}$ (k=0,1,2) to other participants through the classical channel. Obviously, the probability of him randomly obtaining the measurement result ${|k\rangle }_{1}$ is 1/3, and the state of the remaining particles collapses into

(43) $$\begin{array}{c} \begin{array}{cc} |\Omega^{\prime }\rangle & =\prod_{n=1}^{M}{(b_{kn_{0}}|00\rangle }_{A_{n},2n}\alpha_{n}{|0\rangle }_{2n+1}+b_{kn_{1}}\omega^{k}{|01\rangle }_{A_{n},2n}\alpha_{n}{|1\rangle }_{2n+1}\\ & \;\;+b_{kn_{1}}\omega^{2k}{|02\rangle }_{A_{n},2n}\alpha_{n}{|2\rangle }_{2n+1}+b_{kn_{0}}{|10\rangle }_{A_{n},2n}\beta_{n}{|0\rangle }_{2n+1}\\ & \;\;+b_{kn_{1}}\omega^{k}{|11\rangle }_{A_{n},2n}\beta_{n}{|1\rangle }_{2n+1}+b_{kn_{1}}\omega^{2k}{|12\rangle }_{A_{n},2n}\beta_{n}{|2\rangle }_{2n+1}). \end{array} \end{array}$$

**Step 2** The sender Alice skillfully chooses asymmetric bases ${\{|\psi_{kn_{j}\pm }^{\left(n\right)}\rangle }_{A_{n},2n}:j=0,1,2\}$ (n=1,2,⋯,M), which are

(44) $$\begin{array}{c} \begin{array}{cc} |\psi_{kn_{0}\pm }^{\left(n\right)}\rangle_{A_{n},2n} & =\frac{1}{\sqrt{2}}{\left[|00\rangle \pm \left(s_{kn}|11\rangle -t_{kn}|02\rangle \right)\right]}_{A_{n},2n},\\ |\psi_{kn_{1}\pm }^{\left(n\right)}\rangle_{A_{n},2n} & =-\frac{1}{\sqrt{2}}{\left(|01\rangle \pm |12\rangle \right)}_{A_{n},2n},\\ |\psi_{kn_{2}\pm }^{\left(n\right)}\rangle_{A_{n},2n} & \;=\frac{1}{\sqrt{2}}{\left[\left(s_{kn}|02\rangle +t_{kn}|11\rangle \right)\pm |10\rangle \right]}_{A_{n},2n}. \end{array} \end{array}$$

to measure every pair $\left(A_{n},2n\right)$ of her particles for any k=0,1,2 and n=1,2,⋯,M, where real numbers $s_{kn}$ and $t_{kn}$ satisfy

(45) $$\begin{array}{c} s_{kn}=\sqrt{\frac{1}{2}\left(1+\frac{b_{kn_{0}}^{2}}{b_{kn_{1}}^{2}}\right)},\;\;t_{kn}=\sqrt{\frac{1}{2}\left(1-\frac{b_{kn_{0}}^{2}}{b_{kn_{1}}^{2}}\right)}. \end{array}$$

Subsequently, Alice notifies Bob_1_, Bob_2_, ⋯, Bob_*M*_ of the measurement results $|\psi_{k1_{j_{1}}\pm }^{\left(1\right)}\rangle_{A_{1},2}$, $|\psi_{k2_{j_{2}}\pm }^{\left(2\right)}\rangle_{A_{2},4}$, ⋯, $|\psi_{kM_{j_{M}}\pm }^{\left(M\right)}\rangle_{A_{M},2M}$, respectively, through classical channels.

After Alice has measured *M* pairs of particles, the (unnormalized) collapsed states of qutrits 3,5,⋯,2M+1, corresponding to Alice’s measurement results $|\psi_{k1_{j_{1}}\pm }^{\left(1\right)}\rangle_{A_{1},2}$, $|\psi_{k2_{j_{2}}\pm }^{\left(2\right)}\rangle_{A_{2},4}$, ⋯ and $|\psi_{kM_{j_{M}}\pm }^{\left(M\right)}\rangle_{A_{M},2M}$, can be derived by |Φk{jn}±〉=(∏n=1M〈An,2nψknjn±(n)|)|Ω′〉$=\prod_{n=1}^{M}{|\chi_{kn_{j_{n}}\pm }\rangle }_{2n+1},$ where $j_{n}\in \left\{0,1,2\right\}$ and

(46) $$\begin{array}{c} \begin{array}{cc} |\chi_{kn_{0}\pm }\rangle_{2n+1} & =\frac{1}{\sqrt{2}}[\alpha_{n}(b_{kn_{0}}|0\rangle \mp b_{kn_{1}}t_{kn}\omega^{2k}|2\rangle )\;\pm \;\beta_{n}b_{kn_{1}}s_{kn}\omega^{k}{\left|1\rangle \right]}_{2n+1},\\ |\chi_{kn_{1}\pm }\rangle_{2n+1} & =\frac{1}{\sqrt{2}}[\alpha_{n}b_{kn_{1}}\omega^{k}|1\rangle \pm \beta_{n}b_{kn_{1}}\omega^{2k}{\left|2\rangle \right]}_{2n+1},\\ |\chi_{kn_{2}\pm }\rangle_{2n+1} & =\frac{1}{\sqrt{2}}[\alpha_{n}b_{kn_{1}}s_{kn}\omega^{2k}|2\rangle +\beta_{n}(b_{kn_{1}}t_{kn}\omega^{k}|1\rangle \pm b_{kn_{0}}{\left|0\rangle )\right]}_{2n+1}. \end{array} \end{array}$$

It is straightforward to calculate that the probabilities of obtaining the collapsed states $|\chi_{kn_{j_{n}}\pm }\rangle_{2n+1}$ are given by the overlap Pknjn±=〈2n+1χknjn±|χknjn±〉2n+1 as

(47) $$\begin{array}{c} P_{kn_{0}\pm }=P_{kn_{2}\pm }=\frac{1}{4}\left(b_{kn_{0}}^{2}+b_{kn_{1}}^{2}\right)\;\;\mathrm{and}\;\;P_{kn_{1}\pm }=\frac{1}{2}b_{kn_{1}}^{2}. \end{array}$$

**Step 3** After receiving the measurement information from the controller Charlie and the sender Alice, each Bob_*n*_ can perform a local operation, similar to that constructed in Step 3 of Section 3, to obtain the corresponding target state.

We now calculate the overall success probability of the present scheme. First, for a fixed *k* (k=0,1,2), the total probability that the *n*th receiver Bob_*n*_ can successfully obtain the target state is

(48) $$\begin{array}{c} \begin{array}{cc} P_{kn} & =P_{kn_{0}+}+P_{kn_{0}-}+P_{kn_{1}+}+P_{kn_{1}-}+P_{kn_{2}+}+P_{kn_{2}-}\\ & =2(P_{kn_{0}\pm }+P_{kn_{2}\pm }+P_{kn_{1}\pm })\\ & =4\times \frac{1}{4}\left(b_{kn_{0}}^{2}+b_{kn_{1}}^{2}\right)+2\times \frac{1}{2}b_{kn_{1}}^{2}\\ & =b_{kn_{0}}^{2}+2b_{kn_{1}}^{2}=1. \end{array} \end{array}$$

Thus, the total probability that all receivers can successfully obtain their respective target states is $\prod_{n=1}^{M}P_{kn}=1$. Note that Charlie has three possible measurement results, and each result occurs with the same probability of 1/3. Therefore, the overall success probability of the proposed scheme is

$$(\frac{1}{3}\times \prod_{n=1}^{M}P_{kn})\times 3=1,$$

which means that the proposed scheme realizes a perfect controlled multi-output teleportation protocol.

## 6. Discussion and Conclusions

In this paper, we investigate the perfect controlled multi-output quantum teleportation of arbitrary unknown single-qubit states via high-dimensional partially entangled channels. Specifically, a three-dimensional partially entangled five-qutrit state is first constructed as the quantum channel and is pre-shared among the sender, multiple receivers, and the controller. Two unknown single-qubit states are introduced at the sender’s side as the input states. The protocol proceeds as follows. First, the controller performs a single-particle projective measurement and announces the measurement result. Then, according to the Schmidt-coefficient structure of the high-dimensional partially entangled channel, the sender selects the corresponding asymmetric joint measurement basis and performs joint measurements on the relevant particles. Finally, based on the classical information announced by both the controller and the sender, the receivers apply appropriate local unitary recovery operations to reconstruct their respective target quantum states exactly. On this basis, the controlled two-output teleportation scheme based on the three-dimensional partially entangled channel is further generalized to the case of arbitrary *d*-dimensional partially entangled channels. By constructing an asymmetric joint measurement basis in the (2×d)-dimensional Hilbert space and requiring the two largest Schmidt coefficients of the channel to be equal, the collapsed states at the receivers’ side can always preserve the linear structure of the unknown coefficients, thereby enabling perfect state reconstruction. Moreover, the two-output configuration is further extended to the multi-output scenario, allowing a single sender to distribute unknown quantum states to multiple receivers simultaneously within a unified framework. In this way, a unified theoretical framework integrating high-dimensional channels, controlled teleportation, and multi-output quantum information distribution is established.

Compared with existing studies, the novelty of this work is mainly reflected in the following aspects. First, this work extends the class of channel resources available for deterministic perfect quantum teleportation. Conventional perfect quantum teleportation protocols usually rely on ideal maximally entangled channels. However, in practical quantum communication environments, maximally entangled states may degrade into partially entangled states or even mixed states due to environmental noise, decoherence, channel loss, and device imperfections. Therefore, perfect teleportation schemes that rely solely on maximally entangled resources may have limited applicability in practical quantum networks. In contrast, the present scheme directly employs a high-dimensional partially entangled pure state satisfying specific Schmidt-coefficient relations as the quantum channel, and realizes quantum teleportation with both fidelity and success probability equal to 1 under this condition. This shows that, with an appropriate channel structure and measurement design, non-maximally entangled high-dimensional channels can also serve as effective resources for deterministic perfect quantum teleportation.

Second, this work provides a measurement-matching mechanism for achieving both unit fidelity and unit success probability under non-maximally entangled channels. Existing studies have shown that, when non-maximally entangled channels are used, conventional schemes can usually recover the target state with unit fidelity only in certain successful branches, while the overall success probability is less than 1. To improve the success probability, existing schemes often require ancillary particles, entanglement extraction, generalized measurements, additional recovery operations, or repeated transmission processes. Although these methods can improve the transmission success probability to some extent, they usually still retain a success–failure branch structure or require additional quantum resources and multiple rounds of operations. In contrast, the present scheme neither requires the partially entangled channel to be converted into a maximally entangled channel in advance nor relies on postselection or repeated transmission. Instead, it directly exploits the Schmidt-coefficient structure of the high-dimensional partially entangled channel. By constructing an asymmetric joint measurement basis matched with the entanglement structure of the channel, the collapsed states at the receivers’ side corresponding to all measurement outcomes can preserve the linear structure of the coefficients of the unknown input states. Therefore, the receivers only need to perform the corresponding local unitary recovery operations to reconstruct the target states, thereby avoiding the failure branches in conventional probabilistic teleportation.

Third, this work unifies high-dimensional partially entangled resources, a controlled authorization mechanism, and multi-output quantum information distribution within a single protocol framework. High-dimensional quantum channels possess larger Hilbert spaces and more flexible freedom in measurement design, which provides the conditions for constructing asymmetric measurement bases adapted to the structure of partially entangled channels. Meanwhile, the controller’s measurement result is necessary classical information for the receivers to correctly recover the target states, and thus the protocol naturally possesses an authorization-control function. The multi-output structure enables a single sender to distribute unknown quantum states to multiple receivers simultaneously within the same communication process, thereby reducing the possible asynchrony, operational complexity, and low resource utilization associated with conventional sequential transmission schemes. Therefore, the present scheme is not a simple extension of existing single-output teleportation protocols. Instead, it is oriented toward complex multi-user quantum networks and organically combines deterministic transmission, controlled authorization, multi-output parallel distribution, and high-dimensional partially entangled resources.

It should be noted that this paper discusses a typical class of channel-resource degradation in non-ideal quantum communication environments. Ideal quantum teleportation usually relies on maximally entangled channels. However, in practical environments, environmental noise, decoherence, channel loss, and device imperfections may cause maximally entangled states to degrade into non-maximally entangled states, or even further evolve into mixed states. The high-dimensional partially entangled pure-state channel studied in this paper is a typical analytically tractable model among non-maximally entangled resources, characterized by a definite Schmidt-coefficient structure. It does not cover all possible noisy-channel cases. Mixed states generated under general noise usually need to be described by density matrices, and their specific forms depend on the noise type, noise strength, channel dimension, number of particles, and other factors. As a result, their structures are more complicated and difficult to express in a unified form. Therefore, the focus of this paper is to show that, for high-dimensional partially entangled channels with a definite Schmidt-coefficient structure, non-maximally entangled resources can also support perfect quantum teleportation with both fidelity and success probability equal to 1, provided that the measurement basis is matched with the channel structure. At the same time, in practical implementations, non-ideal factors such as decoherence, dephasing, depolarization, amplitude damping, channel loss, imperfect projective measurements, and imperfect local unitary operations during channel preparation, distribution, storage, measurement, and recovery may affect the Schmidt-coefficient structure of the channel and the matching relation between the measurement basis and the channel, thereby reducing the teleportation fidelity or success probability.

From the perspective of security, the proposed scheme has favorable controllable security features. The security of a quantum teleportation protocol first depends on whether the quantum channel can be securely pre-shared among the legitimate participants. Therefore, before the protocol is executed, existing entanglement verification, eavesdropping detection, and participant identity authentication methods in quantum secure communication can be used to check the security of the channel distribution process [62,63,64]. On this basis, the present protocol further introduces the participation of a controller. Even if a receiver obtains the sender’s measurement information, the receiver must also obtain the controller’s measurement result in order to perform the correct local recovery operation and reconstruct the target quantum state. Thus, the controller serves as an authorization center in the protocol and can prevent receivers from independently recovering quantum information without permission. Compared with ordinary uncontrolled teleportation schemes, the present scheme adds a controller-based authorization mechanism on the basis of secure channel distribution, thereby improving the security and controllability of multi-user quantum networks.

In conclusion, this paper focuses on the problem of deterministic perfect quantum teleportation under non-maximally entangled resources and constructs a controlled multi-output quantum teleportation scheme based on high-dimensional partially entangled channels and asymmetric joint measurement bases. Theoretical analysis shows that, when the corresponding Schmidt-coefficient matching conditions are satisfied, the considered high-dimensional partially entangled channel can support perfect quantum teleportation with both fidelity and success probability equal to 1. Meanwhile, the participation of the controller makes the recovery of the target states dependent on authorization information, while the multi-output structure enables multiple receivers to recover their respective quantum states in parallel within the same transmission process. This result provides a theoretical reference for controlled, parallel, and efficient quantum information transmission using non-maximally entangled resources in complex multi-user quantum networks. Future research may further consider noisy environments, mixed-state channels, imperfect measurements, more general Schmidt-coefficient conditions, and experimental resource optimization, so as to promote the development of such protocols toward practical quantum network applications.

## Figures and Tables

**Table 1:** The relationship among Alice’s outcomes, Bob’s operation $U_{kj\pm }^{\left(B\right)}$, Charlie’s operation $U_{kl\pm }^{\left(C\right)}$, and the reconstructed target states on qutrits 3 and 5.

$|\psi_{kj\pm }^{\left(a\right)}\rangle_{A2}$	$U_{kj\pm }^{\left(B\right)}$	Bob’s Target State	$|\psi_{kl\pm }^{\left(b\right)}\rangle_{A^{\prime }4}$	$U_{kl\pm }^{\left(C\right)}$	Charlie’s Target State
$|\psi_{k0\pm }^{\left(a\right)}\rangle_{A2}$	$U_{k0\pm }^{\left(B\right)}$	$\alpha (s_{k}|0\rangle +t_{k}{\left|2\rangle \right)}_{3}+\beta {|1\rangle }_{3}$	$|\psi_{k0\pm }^{\left(b\right)}\rangle_{A^{\prime }4}$	$U_{k0\pm }^{\left(C\right)}$	$x(s_{k}^{\prime }|0\rangle +t_{k}^{\prime }{\left|2\rangle \right)}_{5}+y{|1\rangle }_{5}$
$|\psi_{k1\pm }^{\left(a\right)}\rangle_{A2}$	$U_{k1\pm }$	${\alpha |1\rangle }_{3}+\beta {|2\rangle }_{3}$	$|\psi_{k1\pm }^{\left(b\right)}\rangle_{A^{\prime }4}$	$U_{k1\pm }$	${x|1\rangle }_{5}+y{|2\rangle }_{5}$
$|\psi_{k2\pm }^{\left(a\right)}\rangle_{A2}$	$U_{k2\pm }^{\left(B\right)}$	${\alpha |2\rangle }_{3}+\beta (s_{k}|0\rangle +t_{k}{\left|1\rangle \right)}_{3}$	$|\psi_{k2\pm }^{\left(b\right)}\rangle_{A^{\prime }4}$	$U_{k2\pm }^{\left(C\right)}$	${x|2\rangle }_{5}+y(s_{k}^{\prime }|0\rangle +t_{k}^{\prime }{\left|1\rangle \right)}_{5}$

## Data Availability

All relevant data are included within the manuscript to support the findings of this study.
